# Effects of Cigarette Smoke Exposure on the Gut Microbiota and Liver Transcriptome in Mice Reveal Gut–Liver Interactions

**DOI:** 10.3390/ijms231911008

**Published:** 2022-09-20

**Authors:** Lei Meng, Mengjun Xu, Youwen Xing, Chen Chen, Jiandong Jiang, Xihui Xu

**Affiliations:** Key Laboratory of Agricultural and Environmental Microbiology, Department of Microbiology, College of Life Sciences, Nanjing Agricultural University, Ministry of Agriculture and Rural Affairs, Nanjing 210095, China

**Keywords:** cigarette smoke exposure, gut–liver axis, gut microbiota, lipid metabolism, liver transcriptome

## Abstract

Cigarette smoke exposure has a harmful impact on health and increases the risk of disease. However, studies on cigarette-smoke-induced adverse effects from the perspective of the gut–liver axis are lacking. In this study, we evaluated the adverse effects of cigarette smoke exposure on mice through physiological, biochemical, and histopathological analyses and explored cigarette-smoke-induced gut microbiota imbalance and changes in liver gene expression through a multiomics analysis. We demonstrated that cigarette smoke exposure caused abnormal physiological indices (including reduced body weight, blood lipids, and food intake) in mice, which also triggered liver injury and induced disorders of the gut microbiota and liver transcriptome (especially lipid metabolism). A significant correlation between intestinal bacterial abundance and the expression of lipid-metabolism-related genes was detected, suggesting the coordinated regulation of lipid metabolism by gut microbiota and liver metabolism. Specifically, *Salmonella* (harmful bacterium) was negatively and positively correlated with up- (such as *Acsl3* and *Me1*) and downregulated genes (such as *Angptl4*, *Cyp4a12a*, and *Plin5*) involved in lipid metabolism, while *Ligilactobacillus* (beneficial bacterium) showed opposite trends with these genes. Our results clarified the key role of gut microbiota in liver damage and metabolism and improved the understanding of gut–liver interactions caused by cigarette smoke exposure.

## 1. Introduction

Cigarette smoking is a global pandemic and a major preventable disease worldwide. Although many countries have persisted in advancing tobacco control as a key health priority, more than one billion people around the world still smoke, accounting for more than eight million deaths and costing the global economy USD 1.4 trillion each year [1]. Smoking is primarily associated with respiratory diseases, and it also increases the risk of metabolic-, cardiovascular-, and gastrointestinal-related disorders [2,3,4,5,6]. The toxic components in cigarette smoke are considered to be the biggest factor leading to serious diseases [6]. Among these toxic components, nicotine is the primary active substance of tobacco [7]. Nicotine can be rapidly absorbed from the oral mucosa and respiratory tract, inhaled into the lungs, and rapidly absorbed in the alveoli [8,9,10,11]. In addition, nicotine can also be absorbed through the skin and the gastrointestinal tract [12,13]. Of note, high levels of nicotine have been found in gastric juice, with nicotine concentrations of >800 ng/mL in gastric juice [13,14].

The gut microbiota is considered to be the “second genome” of human beings [15]. The composition and stability of gut microbiota are crucial to human health because the imbalance of gut microbiota is usually associated with disease [16,17]. For example, *Muribaculum* is associated with the regulation of body weight [18]; *Alistipe* plays a critical role in colitis [19]; a reduced abundance of *Akkermansia* might lead to a thinner mucous layer and impaired intestinal barrier integrity [20]. It has been shown that probiotics, which are living microorganisms that confer a health benefit to the host, can regulate the gut microbiota to improve the gut microenvironment [21,22]. For example, probiotics may have some positive effects in the treatment of inflammatory bowel disease (IBD) by regulating gut microbiota [23]; *Lactobacillus acidophilus* NCFM, as a probiotic, can affect gene expression involved in the formation of mucus-binding proteins and adhesion factors in the process of intestinal transport, driving gut fitness [24]. In addition, fecal microbiota transplantation (FMT), which is an emerging therapy, can also induce alterations in the gut microbiota. It was first used to treat refractory *Clostridium difficile* infection [25]. Therefore, the gut microbiota plays an important role in maintaining the stability of the intestinal environment and regulating host gene expression [26].

The gut microbiota is susceptible to environmental factors, including smoking. For example, recent studies have indicated that cigarette smoking promotes shifts in gut microbial communities [7,27,28,29]. Nicotine is the main active substance of tobacco, and a large amount of evidence accumulated from animal and human studies supports the view that nicotine affects the gut microbiota. For examples, cigarette smoke exposure increased the relative abundance of *Clostridium*, while it decreased the relative abundance of *Lactoccoci*, *Ruminococcus*, and Enterobacteriaceae compared to control mice [30]; Chi et al. [7] reported that the oral administration of nicotine altered the gut microbiota; Lee et al. [28] reported that cigarette smoking increased Bacteroidetes and decreased Firmicutes and Proteobacteria in smokers compared with never-smokers. It was reported that expression changes in oxidative-stress-related enzymes in mice exposed to cigarette smoke were the cause of gut ecological imbalance [30]. In addition, long-term exposure to cigarette smoke can promote tissue hypoxia, ischemia, and mucosal inflammation of the gastrointestinal tract; lead to intestinal barrier dysfunction; and further contribute to a dysfunctional gut environment [4,31]. In addition, the toxic components in cigarette smoke could reduce the antibacterial effect of intestinal antimicrobial peptides, change the intestinal microenvironment, and induce gut microbiota dysbiosis [32]. However, the potential mechanism between cigarette smoking and gut microbiota disorders is still unclear.

The liver is the main organ regulating host metabolism [33]. The bidirectional relationship between the gut microbiota and the liver is called the gut–liver axis [34]. In recent years, the gut–liver axis has attracted considerable attention. For example, the gut microbiota can be involved in the pathogenesis of many chronic liver diseases, such as alcoholic liver disease [35], nonalcoholic fatty liver disease [36], and cirrhosis [37]. However, until now, in the context of cigarette smoke exposure, the role of gut microbiota dysbiosis in liver gene expression and liver damage is still largely unknown.

**Figure 1:**
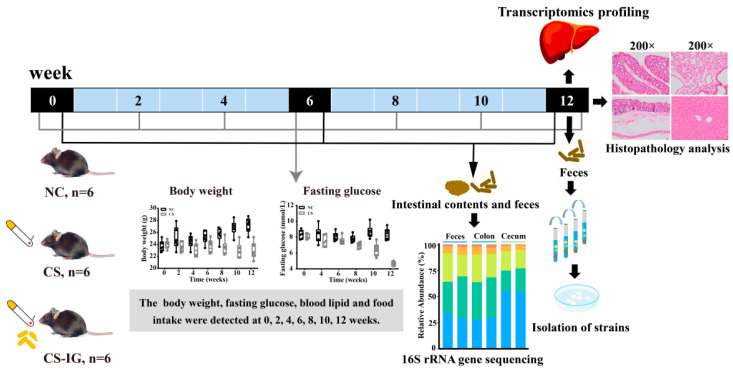
Schematic representation of the experimental design. Mice were treated with normal air exposure (NC group, *n* = 6) or cigarette smoke exposure (CS group, *n* = 6). In addition, mice were intragastrically administered normal saline without bacteria (NC and CS group, *n* = 6) or with strain JQ581 (CS-IG group, *n* = 6). After two weeks of environmental adaptation, mice with similar body weights and blood sugar and lipid indices were selected and grouped randomly to ensure that the state of the mice before the experiment was consistent. The mice in the CS and CS-IG groups were exposed to 2 cigarettes at an air flow rate of 3 L/min for 20–30 min at a time and exposed three times a day. The treatment lasted for 12 weeks.

In this study, the effects of cigarette smoking on gut microbiota disorders and liver damage are studied. Specifically, mice are exposed to cigarette smoke for 12 weeks, and physiological and biochemical indicators, such as body weight, blood sugar, and blood lipids, are monitored during cigarette smoke exposure (Figure 1 and Figure S1). The influence of cigarette smoke exposure on the gut microbiota in mice is analyzed through 16S rRNA gene sequencing (Figure 1). Moreover, transcriptome sequencing is used to analyze differential gene expression in the liver to identify the related genes or pathways and potential molecular mechanisms that lead to liver function injury under cigarette smoke exposure. In addition, correlations between alterations in gut microbiota and liver differential gene expression are analyzed to explore the potential relationship between gut microbiota disorders and liver function injury induced by cigarette smoke in mice. Furthermore, we also perform an experiment in which mice are intragastrically administered the nicotine-degrading bacterium *Pseudomonas putida* JQ581 [38] to alleviate the damage caused by cigarette smoke exposure (Figure 1). There are no known strains of *P. putida* that are animal or plant pathogens, thus *P. putida* are considered to be environmentally innocuous [39]. Our results are expected to improve our understanding of gut microbiota dysbiosis induced by cigarette smoke exposure, as well as the interactions between gut microbiota and liver gene expression under the context of cigarette smoke exposure.

## 2. Results

### 2.1. Exposure to Cigarette Smoke Changed Physiological and Biochemical Indicators in Mice

To explore the influence of cigarette smoke exposure on mice, the mice were treated with normal air exposure (NC group as control) or cigarette smoke exposure (CS group). In addition, treatment with a combination of cigarette smoke exposure and intragastric administration of the nicotine-degrading strain JQ581 (CS-IG group) was used to explore the potential of intragastric administration of the nicotine-degrading strain to improve mouse health (Figure 1).

**Figure 2:**
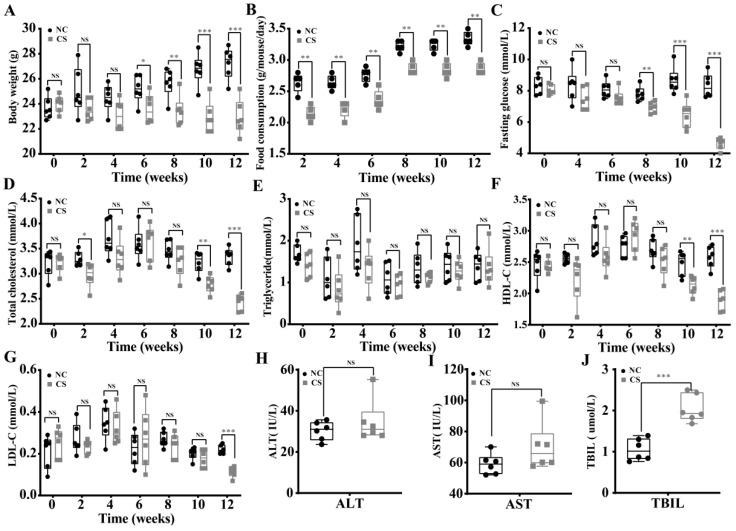
Effects of cigarette smoke exposure on the physiological and biochemical indicators of mice. (**A**) Body weight. (**B**) Food consumption. (**C**) Fasting glucose. (**D**) Serum total cholesterol. (**E**) Serum triglyceride. (**F**) Serum HDL-C. (**G**) Serum LDL-C. (**H**–**J**) AST (**H**) and ALT (**I**) activities and TBIL (**J**) contents in blood serum. LDL-C, low-density lipoprotein cholesterol; HDL-C, high-density lipoprotein cholesterol; AST, aspartate aminotransferase; ALT, alanine aminotransferase; TBIL, total bilirubin. NC, mice treated with normal air exposure; CS, mice treated with cigarette smoke exposure. Comparison of means between groups was performed with Student’s *t*-test. (*, *p* < 0.05; **, *p* < 0.01; ***, *p* < 0.001; NS, not significant; *n* = 6).

After 12 weeks of exposure to cigarette smoke, the body weights of mice from the CS group were significantly lower than those of mice from the NC group (*p* < 0.05, Figure 2A). Consistently, although the food consumption of mice from both the NC and CS groups increased from 2 to 12 weeks, the food consumption of the CS group was significantly lower than that of the NC group (*p* < 0.05, Figure 2B). Notably, after a week of treatment, we observed behavioral changes between treated and control mice. We found that mice from the CS group were more active at night and more irritable during daytime compared to those from the NC group. In addition, the blood glucose concentrations of mice from the CS group progressively decreased over the 12-week period, leading to hypoglycemia (Figure 2C). In contrast, relatively stable concentrations of blood glucose in the NC group were detected, which were significantly higher than those in the CS group (*p* < 0.05, Figure 2C). The serum lipid profiles showed that the content of serum lipids was significantly decreased after cigarette smoke exposure for 12 weeks (*p* < 0.05, Figure 2D–G), as revealed by the decreased levels of serum total cholesterol (TC, Figure 2D), high-density lipoprotein cholesterol (HDL-C, Figure 2F), and low-density lipoprotein cholesterol (LDL-C, Figure 2G). The level of serum triglyceride (TG) in the CS group was not significantly different from that in the NC group (Figure 2E).

To evaluate the effects of cigarette smoke exposure on liver function in mice, biochemical indices, including aspartate aminotransferase (AST), alanine aminotransferase (ALT), and total bilirubin (TBIL), in blood serum were measured at 12 weeks after cigarette smoke exposure (Figure 2H–J). The obtained results showed that the serum TBIL content was significantly increased in the CS group compared to the NC group (*p* < 0.05, Figure 2J). In addition, cigarette smoke exposure also led to slight increases in the serum AST and ALT levels in the CS group (Figure 2H,I). These results showed that exposure to cigarette smoke could cause some degree of liver damage in mice. Meanwhile, hematoxylin and eosin (H&E) staining of liver tissue showed no significant pathological changes after 12 weeks of cigarette smoke exposure (Figure S2), and similar results were detected for the cecum, colon, and lungs (Figure S2). This result suggested that, on the surface, cigarette smoke exposure did not cause remarkable physical damage to the liver, cecum, colon, or lungs during the time period of 12 weeks. Overall, these results suggested that cigarette smoke exposure provoked changes in physiological and biochemical indicators in mice, indicating lipid metabolism disorders and liver damage in mice.

Of note, the physiological and biochemical indicators of the CS-IG group were similar to those of the CS group (Figure S3). To assess the colonization of strain JQ581 in mice, we isolated strain JQ581 from the fecal samples of mice from the CS-IG group at different times after intragastric administration (Figure S4). The counts of the isolated strain JQ581 sharply decreased, and strain JQ581 was not detectable at 12 h after treatment (Figure S4). These results showed that the health of mice exposed to cigarette smoke was not improved by intragastric administration of the nicotine-degrading strain JQ581, which may be caused by failure of colonization of strain JQ581 in mice.

### 2.2. Exposure to Cigarette Smoke Caused Disorders in the Gut Microbiota

**Figure 3:**
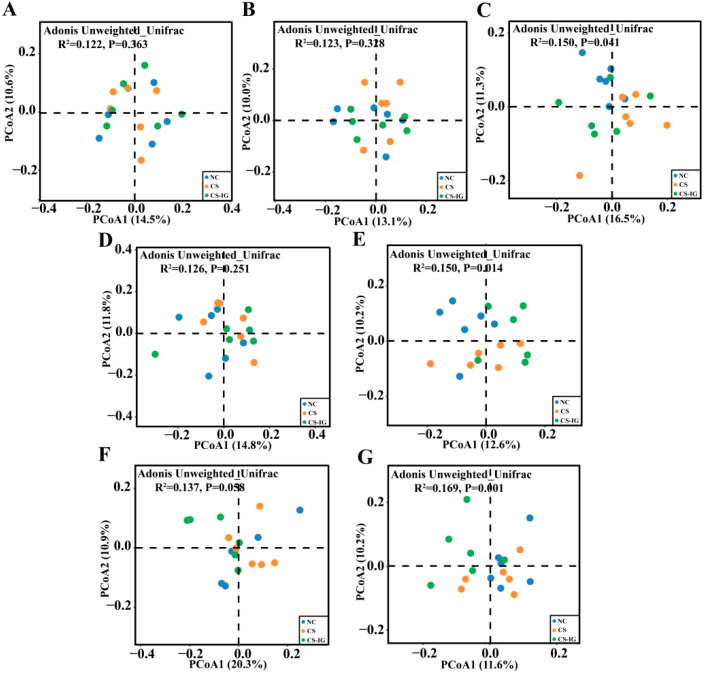
The effects of cigarette smoke exposure and intragastric administration of strain JQ581 on the structure of gut microbiota. Principal coordinate analyses (PCoAs) based on ASVs of feces, colon, and cecal contents in mice are shown. (A-C) PCoAs of feces collected at 0 (**A**), 6 (**B**), and 12 (**C**) weeks after treatment. (**D**,**E**) PCoAs of colon collected at 6 (**D**) and 12 (**E**) weeks after treatment. (**F**,**G**) PCoAs of cecal contents collected at 6 (**F**) and 12 (**G**) weeks after treatment. NC, mice treated with normal air exposure; CS, mice treated with cigarette smoke exposure; CS-IG, mice treated with combination of cigarette smoke exposure and intragastric administration of nicotine-degrading strain JQ581.

The gut microbiota in the feces, colon, and cecal contents of mice from the NC, CS, and CS-IG groups were analyzed using 16S rRNA gene sequencing. The coverage index was 1 in all three groups, indicating that the sequencing depth met the requirements for subsequent analysis (Table S1). The ACE, Chao1, and Shannon indices were used to evaluate the impacts of cigarette smoke exposure on the α diversity of the gut microbiota (Table S1). Generally, the obtained results showed that there was no significant difference in the α diversity of the gut microbiota among the three treatments at 6 or 12 weeks (Table S1). Similarly, after 6 weeks of treatment, no significant differences in community structure among the NC, CS, and CS-IG groups were detected (Figure 3A,B,D,F). However, after 12 weeks of treatment, significant differences in community structure among the NC, CS, and CS-IG groups were detected (Figure 3C,E,G). Notably, significant separations were detected in the comparisons of NC vs. CS and NC vs. CS-IG rather than CS vs. CS-IG (Figure S5). These results showed that cigarette smoke exposure induced remarkable changes in the gut microbiota community structure. In addition, the similar community structures between the CS and CS-IG groups indicated that the alteration in community structure was mainly driven by cigarette smoke exposure rather than the intragastric administration of strain JQ581. The limited effect of the intragastric administration of strain JQ581 on gut microbiota was also consistent with the result of no improvement in the health of mice exposed to cigarette smoke through the intragastric administration of strain JQ581.

**Figure 4:**
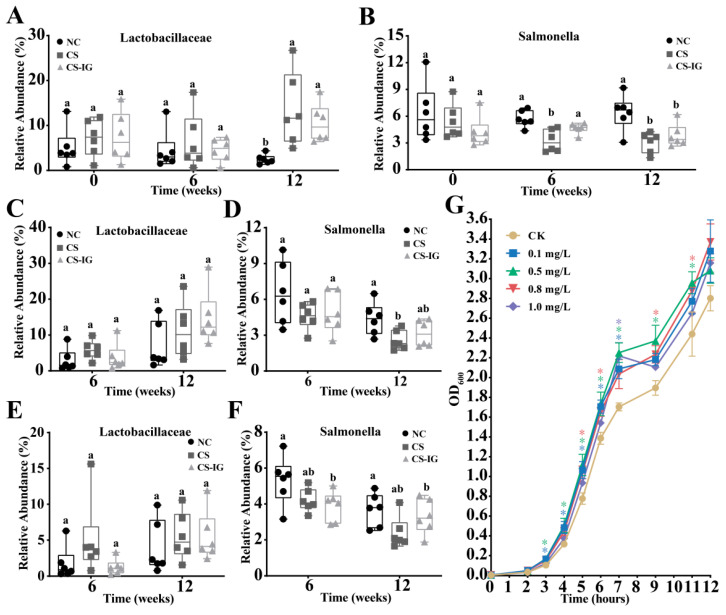
The relative abundance of Lactobacillaceae and *Salmonella* in gut microbiota of mice: (**A**,**B**) fecal, (**C**,**D**) colon, and (**E**,**F**) cecal contents. (**G**) Effects of different concentrations of nicotine on the growth of *Limosilactobacillus* sp. LM1. The strain LM1 was isolated from both feces and intestinal contents of mice. NC, mice treated with normal air exposure; CS, mice treated with cigarette smoke exposure; CS-IG, mice treated with combination of cigarette smoke exposure and intragastric administration of nicotine-degrading strain JQ581. Different letters indicate significant differences (Duncan’s test, *n* = 6, *p* < 0.05). Asterisks indicate significant differences (Duncan’s test, *n* = 3, *p* < 0.05).

The compositions of microbiota from the feces, colon, and cecal contents were analyzed at both the phylum and genus levels (Figure S6). At the phylum level, the gut microbiota mainly consisted of Firmicutes, Bacteroidetes, Proteobacteria, and Verrucomicrobia for all the samples (Figure S6). At the genus level, the predominant genera (top ten) were *Muribaculum*, *Cronobacter*, *Eisenberglella*, *Ligilactobacillus*, *Salmonella*, *Vibrio*, *Alistipes*, *Akkermansia*, *Duncaniella*, and *Lachnoclostridium* (Figure S6). We then focused on the relative abundance of *Salmonella* (a common harmful bacterium) and Lactobacillaceae (a common probiotic). For the microbiota from feces, no significant differences in the abundance of Lactobacillaceae or *Salmonella* were detected at 0 and 6 weeks after treatment among the NC, CS, and CS-IG groups (Figure 4A,B). However, we observed a significant increase in the abundance of Lactobacillaceae at 12 weeks after treatment in CS (or CS-IG) compared to NC, while the abundance of *Salmonella* was significant decreased at 12 weeks after treatment (Figure 4A,B). Similar results were detected in the microbiota from the colon and cecal contents (Figure 4C–F). To test whether cigarette smoke exposure increased the growth of strains in Lactobacillaceae, we isolated a strain, *Limosilactobacillus* sp. LM1 (Figure S7), and analyzed the growth of strain LM1 under different concentrations of nicotine (Figure 4G). The results showed that nicotine (0.1–1.0 mg/L) significantly promoted the growth of stain LM1. These results were consistent with the increased abundance of Lactobacillaceae in gut microbiota treated with cigarette smoke exposure. 

**Figure 5:**
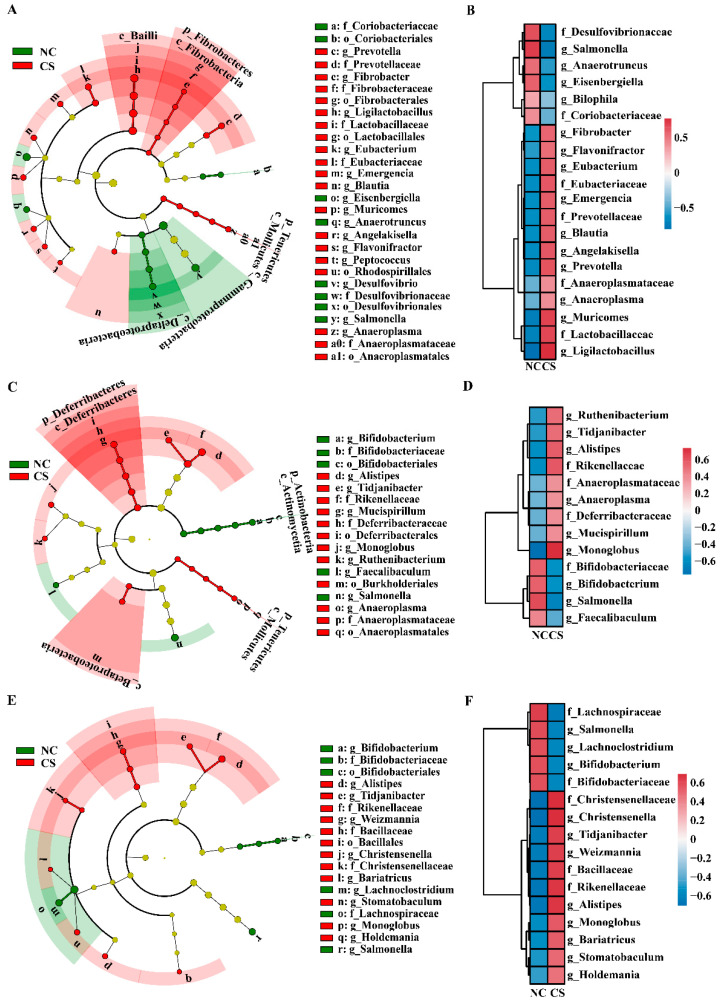
Taxonomic variation in the gut microbiota induced by cigarette smoke exposure. (**A**,**B**) Cladogram of LEfSe analysis (LDA > 3) of microbiota from feces (**A**) and heatmap showing relative abundances of differentially abundant genera and families identified through LEfSe analysis of feces (**B**). (**C**,**D**) Cladogram of LEfSe analysis (LDA > 3) of microbiota from colon (**C**) and heatmap showing relative abundances of differentially abundant genera and families identified through LEfSe analysis of colon (**D**). (**E**,**F**) Cladogram of LEfSe analysis (LDA > 3) of microbiota from cecal contents (**E**) and heatmap showing relative abundances of differentially abundant genera and families identified through LEfSe analysis of cecal contents (**F**). Different color nodes indicate microbial communities that are significantly enriched in corresponding groups and significantly different between groups. The graph of evolutionary branches in the cladogram plots from the center outward represents domain, kingdom, phylum, class, order, family, and genus at the taxonomic level. The size of each node that represents a species at each classification level is positively correlated with the abundance of the species. The yellow nodes represent species without significant difference.

To identify the specific bacterial taxa (biomarkers) influenced by cigarette smoke exposure, the microbiota compositions were compared between the NC and CS groups through a LEfSe analysis (Figure 5). At the family or genus level, a total of 22 bacterial taxa that differed in abundance between the two groups from the feces were identified (Figure 5A,B). Specifically, the relative abundances of f_Anaeroplasmataceae, f_Eubacteriaceae, f_Fibrobacteraceae, f_Lactobacillaceae, f_Prevotellaceae, g_*Anaeroplasma*, g_*Angelakisella*, g_*Blautia*, g_*Emergencia*, g_*Eubacterium*, g_*Fibrobacter*, g_*Flavonifractor*, g_*Ligilactobacillus*, g_*Muricomes*, g_*Peptococcus*, and g_*Prevotella* were significantly elevated in the CS group compared to the NC group, while the relative abundances of f_Coriobacteriaceae, f_Desulfovibrionaceae, g_*Anaerotruncus*, g_*Desulfovibrio*, g_*Eisenbergiella*, and g_*Salmonella* were significantly decreased (Figure 5B). For the microbiota from the colon, 13 bacterial taxa that differed in abundance between the NC and CS groups were identified at the family or genus level (Figure 5C,D). Among them, the relative abundances of f_Anaeroplasmataceae, f_Deferribacteraceae, f_Rikenellaceae, g_*Alistipes*, g_*Anaeroplasma*, g_*Monoglobus*, g_*Mucispirillum*, g_*Ruthenibacterium*, and g_*Tidjanibacter* were significantly increased in the CS group, while those of f_Bifidobacteriaceae, g_*Bifidobacterium*, g_*Faecalibaculum*, and g_*Salmonella* were significantly decreased (Figure 5D). Similarly, for microbiota from cecal contents, 16 bacterial taxa that differed in abundance between the NC and CS groups were identified at the family or genus level (Figure 5E,F). The relative abundances of f_Bacillaceae, f_Christensenellaceae, f_Rikenellaceae, g_*Alistipes*, g_*Bariatricus*, g_*Christensenella*, g_*Holdemania*, g_*Monoglobus*, g_*Stomatobaculum*, g_*Tidjanibacter*, and g_*Weizmannia* were significantly increased in the CS group, while those of f_Bifidobacteriaceae, f_Lachnospiraceae, g_*Bifidobacterium*, g_*Lachnoclostridium*, and g_*Salmonella* were significantly decreased (Figure 5F). Combined with the obtained results of the PCoA, these results indicated that cigarette smoke exposure induced remarkable compositional and structural shifts in the gut microbiota of mice, which might further influence the metabolism and health of mice.

### 2.3. Exposure to Cigarette Smoke Altered the Liver Transcriptome of Mice

To further explore the effects of cigarette smoke exposure on the livers of mice, a transcriptomic analysis was performed on liver samples from the NC, CS, and CS-IG groups. After sequencing and filtering, the Q30 of high-quality sequences was greater than 91.99%, and the average GC content was 48.64% (Table S2). The high-quality sequences were mapped to the mouse reference genome (version GRCm39), with a mapping rate of 87.67–92.67% (Table S2). These results indicated that the reference genome was appropriate, and the sequencing result was of high quality and could be used for further analyses.

**Figure 6:**
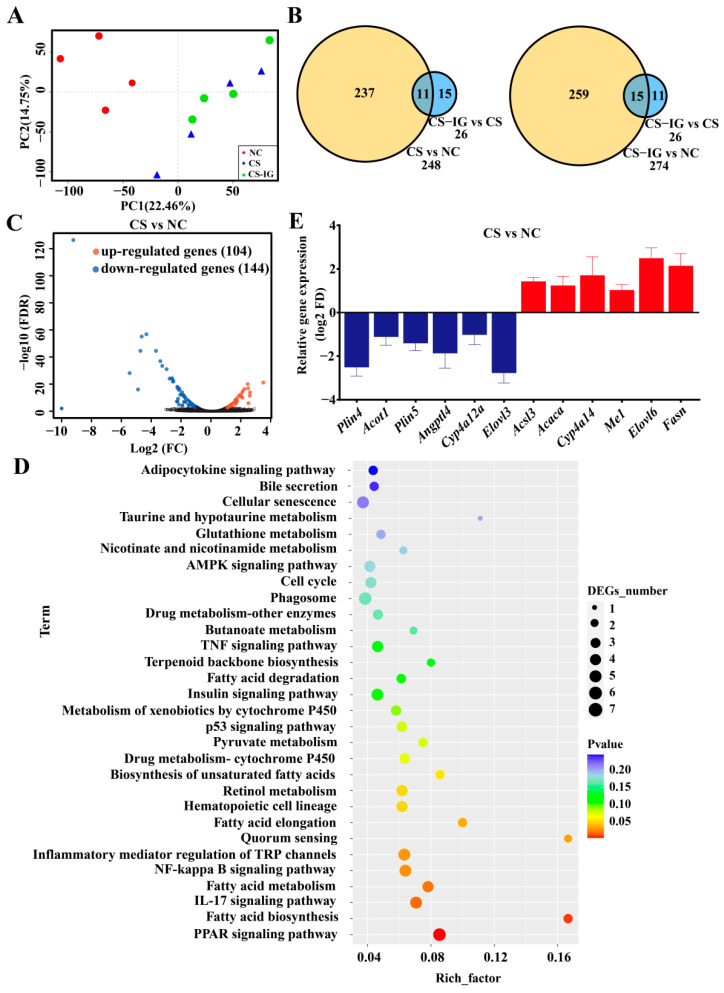
Effects of cigarette smoke exposure on the liver transcriptome of mice. (**A**) Principal component analysis (PCA). (**B**) Differentially expressed genes (DEGs) detected among NC, CS, and CS-IG groups. (**C**) Volcano diagram of DEGs. (**D**) KEGG enrichment pathway analysis. (**E**) DEGs involved in the lipid metabolism pathway. Data were expressed as means ± SEM (*n* = 4). NC, mice treated with normal air exposure; CS, mice treated with cigarette smoke exposure; CS-IG, mice treated with combination of cigarette smoke exposure and intragastric administration of nicotine-degrading strain JQ581.

A principal component analysis (PCA) based on the transcriptome profiles showed segregation of the NC and other two groups (CS and CS-IG), with no segregation between the CS and CS-IG groups (Figure 6A). A total of 22,174 genes were identified in the two treatment groups. Genes with a fold change ≥ 2 and a *p*-value < 0.05 were considered differentially expressed genes (DEGs). All the *p*-values were corrected with a false discovery rate (FDR) based on multiple hypothesis testing using the Benjamini–Hochberg procedure. Compared with the NC group, there were 248 DEGs in the CS group (Figure 6B). In contrast, only 26 DEGs were detected in the comparison of CS-IG vs. CS (Figure 6B). These results showed that CS treatment induced remarkable differences in gene expressions compared to NC, and the CS and CS-IG treatments showed similar influences on gene expressions. Thus, we focused on the 248 DEGs in the comparison of CS vs. NC, including 104 upregulated genes and 144 downregulated genes (Figure 6C).

To further analyze the function of these DEGs, annotation of the DEGs based on gene ontology (GO) was performed, and they were classified into three main types, including biological processes, cellular components, and molecular functions (Figure S8). The obtained results showed that cigarette smoke exposure mainly influenced the GO categories of biological regulation, cellular process, metabolic process, multicellular organismal process, and response to stimulus, which were all associated with the growth and management of hepatocytes. GO enrichment analyses revealed that the DEGs were involved in “lipid metabolism”, “insulin regulation”, “response to organic substance”, “response to nutrient levels”, and “circadian rhythm” (Figure S9).

An enrichment analysis based on the Kyoto Encyclopedia of Genes and Genomes (KEGG) was also performed to determine the associations of DEGs with metabolic pathways (Figure 6D). The obtained results showed that eight metabolic pathways were affected by cigarette smoke exposure (Figure 6D). In particular, the DEGs were mainly involved in four metabolic pathways related to liver lipid metabolism, including fatty acid biosynthesis, fatty acid elongation, fatty acid metabolism, and the PPAR signaling pathway (Figure 6D). Specifically, 12 lipid-metabolism-related genes were significantly up- or downregulated in CS compared to NC samples (Figure 6E). Together, these results indicated that cigarette smoke exposure could affect liver lipid metabolism in mice. In addition, cigarette smoke exposure also led to the significant downregulation of genes in some pathways associated with the liver immune response (Figure S10).

### 2.4. Correlation of Cigarette-Smoke-Exposure-Induced Gut Microbiota Dysbiosis with DEGs in the Liver

**Figure 7:**
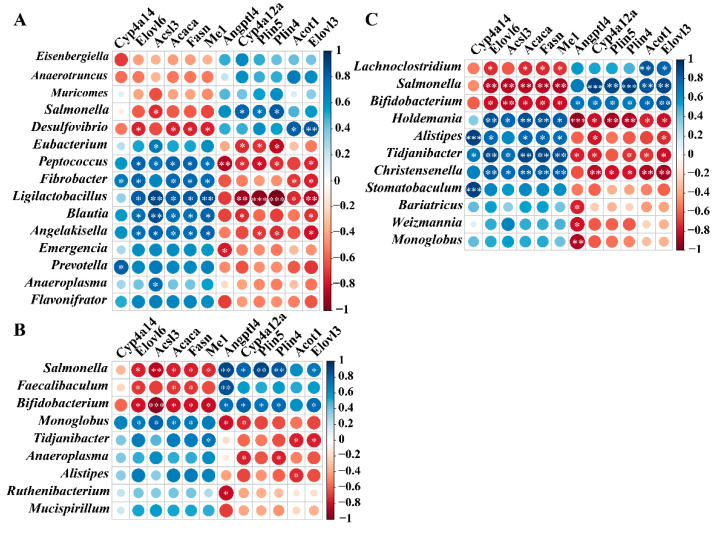
Interactions between gut microbiota and the expressions of genes related to lipid metabolism in the liver. (**A**–**C**) Spearman correlations of gut microbiota from fecal (**A**), colon (**B**), and cecal contents (**C**) with genes involved in lipid metabolism in the liver. Blue and red represent positive and negative correlations, respectively; * *p* < 0.05; ** *p* < 0.01; *** *p* < 0.001; *n* = 4.

To investigate the relationships between gut microbiota and liver gene expression, a correlation analysis was performed between gut microbiota dysbiosis and DEGs in the liver under the context of cigarette smoke exposure. We screened 12 and 10 genes associated with lipid metabolism and immune response from the DEGs, respectively (Figure 6E and Figure S10). In addition, 15, 9, and 11 genera with significant differences in the comparison of CS vs. NC were selected in the fecal, colon, and cecal contents, respectively (Figure 5). Then, we performed a Spearman correlation analysis and visualized the correlation between the relative abundance of bacteria and gene expression in the liver (Figure 7 and Figure S11).

For the feces, the abundances of *Peptococcus* and *Ligilactobacillus* strongly correlated with the expressions of lipid-metabolism-related genes (≥10 genes), while *Desulfovibrio*, *Fibrobacter*, *Blautia*, and *Angelakisella* were correlated with most of the lipid-metabolism-related genes (≥6 genes, Figure 7A). Interestingly, *Peptococcus*, *Fibrobacter*, *Ligilactobacillus*, *Blautia*, and *Angelakisella* were positively associated with *Cyp4a14*, *Elovl6*, *Acsl3*, *Acaca*, *Fasn*, and *Me1* and were negatively associated with *Angptl4*, *Cyp4a12a*, *Plin5*, Plin4, *Acot1*, and *Elovl3* (Figure 7A). In contrast, *Desulfovibrio* showed opposite trends of association with these genes (Figure 7A). For the colon contents, we observed significant gene–bacteria correlations of *Salmonella* and *Bifidobacterium* with lipid-metabolism-related genes (≥10 genes), including positive correlations for *Angptl4*, *Cyp4a12a*, *Plin5*, *Plin4*, and *Elovl3* and negative correlations for *Elovl6*, *Acsl3*, *Acaca*, *Fasn*, and *Me1* (Figure 7B). In addition, *Monoglobus* was positively associated with *Elovl6*, *Acsl3*, *Acaca*, and *Fasn* and negatively associated with *Angptl4* and *Cyp4a12a* (Figure 7B). For the cecal contents, *Salmonella*, *Bifidobacterium*, *Holdemania*, *Tidjanibacter*, and *Christensenella* strongly correlated with lipid-metabolism-related genes (≥10 genes), while *Lachnoclostridium* and *Alistipes* were correlated with most lipid-metabolism-related genes (≥6 genes, Figure 7C). Among them, *Holdemania*, *Alistipes*, *Tidjanibacter*, and *Christensenella* were positively associated with *Cyp4a14*, *Elovl6*, *Acsl3*, *Acaca*, *Fasn*, and *Me1* and were negatively associated with *Angptl4*, *Cyp4a12a*, *Plin5*, *Plin4*, *Acot1*, and *Elovl3* (Figure 7C). However, *Salmonella* and *Bifidobacterium* showed opposite trends of association with these genes (Figure 7C).

In addition, we also explored the potential relationship between DEGs involved in the immune response in liver tissues and differentially abundant bacteria (Figure S11). In the feces of mice, the genera *Salmonella* and *Desulfovibrio* presented positive correlations with immune-response-related genes (Figure S11A). *Peptococcus*, *Ligilactobacillus*, *Angelakisella*, *Prevotella*, *Fibrobacter*, *Flavonifrator*, and *Anaeroplasma* were the main genera that presented negative correlations with immune-response-related genes (Figure S11A). In the colon contents of mice, we observed significantly positive correlations of *Salmonella* and *Bifidobacterium* with immune-response-related genes (Figure S11B), and *Monoglobus* showed significantly negative correlations with immune-response-related genes (Figure S11B). In the cecal contents of mice, *Salmonella* and *Bifidobacterium* were the main microbiota constituents that presented positive correlations with immune-response-related genes (Figure S11C). In contrast, *Christensenella*, *Holdemania*, *Alistipes*, and *Tidjanibacter* were strongly negatively correlated with immune-response-related genes (Figure S11C). Together, these correlations indicated that changes in the gut microbiota of mice exposed to cigarette smoke were closely related to the expressions of liver genes and disease development. Of note, in both colon and cecal contents, *Salmonella* and *Bifidobacterium* showed a significant correlation with both lipid-metabolism-related and immune-response-related genes, indicating that these two genera could be biomarkers for liver injury induced by cigarette smoke exposure in mice.

## 3. Discussion

In this study, we systematically studied the effects of cigarette smoke exposure on the gut microbiota and liver transcriptome in mice and established a connection between the intestine and liver. We showed that cigarette smoke exposure could induce health damage in mice by inducing gut microbial disorder and disrupting liver metabolism. Our results not only provided theoretical support for the potential risk and related diseases of smoking but also improved the understanding of the gut–liver interactions caused by cigarette smoke exposure. In addition, these findings provided important information for identifying potential biomarkers for cigarette-smoke-induced health damage.

We showed that cigarette smoke exposure significantly reduced the body weights of mice compared to the control group, which is consistent with previous studies [40,41,42,43]. In addition, we also found that the food intake of mice exposed to cigarette smoke significantly decreased compared with that of the control group. In agreement with these findings, nicotine, as the main toxic component in tobacco, has been shown to induce weight loss by decreasing appetite and reducing food intake [43,44]. In addition, feeding mice a tobacco mixture or nicotine could lead to weight loss by stimulating adipose lipolysis [45]. Consistently, we found that the contents of serum lipids, including serum TC, HDL-C, and LDL-C levels, were significantly decreased after cigarette smoke exposure in mice. In addition, GO and KEGG enrichment analyses showed that “lipid metabolism” was enriched in the CS group, indicating that cigarette smoke exposure led to disordered lipid metabolism in mice. Of note, gut microbiota disorders may have a negative impact on body weight changes [46]. We found that the abundance of the bacterial families Christensenellaceae and Rikenellaceae significantly increased after cigarette smoke exposure. It has been reported that Christensenellaceae was enriched in individuals with low body mass index [47] and associated with healthy BMI values and reduced diet-induced weight gain [48,49]. Alard et al. [50] showed that the abundance of Rikenellaceae was negatively correlated with body weight. In addition, we found that the abundance of intestinal Lactobacillaceae significantly increased after cigarette smoke exposure, which is consistent with a previous study showing that lactobacilli could limit body weight gain in obese mice [51]. Together, these results show that cigarette smoke exposure induced body weight loss in mice by reducing food intake and disorders of both the gut microbiota and liver metabolism, especially lipid metabolism.

Lipid metabolism disorder is one of the signals of liver injury [33,52]. The serum biomarkers most commonly used to detect liver injury are serum ALT, AST, and TBIL levels [53]. Our results showed that the contents of serum TBIL were significantly increased in the CS group. Although no significant differences in serum ALT and AST levels were detected in the CS group, the contents of serum ALT and AST in the CS group were higher than those in the NC group. Together, these results indicated that cigarette smoke exposure for 12 weeks induced damage to liver function in mice. The damage was also revealed by the alteration in the transcriptome profiles of liver tissue under cigarette smoke exposure. A PCA showed segregation of the liver transcriptome between the NC and CS groups. In addition, we also observed enrichment in liver DEGs in lipid-metabolism-related pathways, such as upregulation of *Acsl3* and *Cyp4a14* and downregulation of *Elovl3*. Specifically, *Elovl3* is a gene involved in the synthesis of saturated and monounsaturated long-chain fatty acids that maintains lipid homeostasis by supplementing the intracellular triacylglycerol pool [54,55]. The downregulation of *Elovl3* in the CS group indicated that lipid synthesis in the liver was impaired. The upregulation of *Cyp4a14* has been proved to be involved in the PPAR signaling pathway and is related to fatty acid oxidative stress and lipid peroxidation, resulting in nonalcoholic fatty liver disease, which is one of the most common chronic liver diseases in the world [56,57]. *Acsl3* is a member of the long-chain acyl CoA synthase family (ACSLs) that converts free long-chain fatty acids into fatty acyl-CoA esters for lipid synthesis and β-oxidation. *Acsl3* increases the proliferation, migration, and invasion of tumor cells by increasing fatty acid β-oxidation, which is conducive to the occurrence of malignant tumors [58,59]. Therefore, the upregulation of *Acsl3* in the CS group suggested that cigarette smoke exposure could increase the risk of cancer.

Our results showed that cigarette smoke exposure significantly changed the composition and structure of gut microbiota in mice. Specifically, cigarette smoke exposure led to an increase in the relative abundance of *Eubacterium* and Lactobacillaceae and a decrease in the relative abundance of Lachnospiraceae. It was shown that cholesterol could be metabolized by *Eubacterium* [60]. In addition, oral administration of the probiotic *Lactobacillus* or its mixture in mice could eliminate high-fat-diet-related liver steatosis and dyslipidemia [50], which illustrates that *Lactobacillus* has the characteristics of reducing cholesterol. Furthermore, the abundance of Lachnospiraceae was positively correlated with plasma HDL-C [61]. These results suggest that alterations in the gut microbiota might be involved in the regulation of lipid metabolism under cigarette smoke exposure. Consistently, it was shown that short-chain fatty acids (SCFAs) were the main fermentation products from substrates broken down by the gut microbiota, which play a vital role in actively regulating host fat storage [62]. In addition, SCFAs can reduce plasma cholesterol and inhibit liver cholesterol synthesis [63,64]. Considering that the DEGs in liver tissues were mostly involved in lipid metabolism, these results suggest the coordinated regulation of lipid metabolism by the gut microbiota and liver metabolism under cigarette smoke exposure.

In this study, we showed that cigarette smoke exposure triggered decreased abundances in Salmonella and increased abundances in Lactobacillaceae. Notably, it is not safe to conclude benefits of smoking by decreased Salmonella and increased Lactobacillaceae. Firstly, the abundance changes in the two bacteria might be not enough to induce benefits. Secondly, the stability of gut microbiota is crucial for health rather than the two bacteria only. Thirdly, the influences of nicotine are systemic, which are not limited to gut; thus, the benefits (if any) to the gut microbiota might be overwhelmed by other adverse effects.

We observed a significant increase in Deferribacteraceae and *Mucispirillum* in the CS group, while *Salmonella* was significantly decreased in the CS group. *Mucispirillum schaedleri*, the sole known representative of Deferribacteraceae present, could compete for nutrients and had a beneficial effect on *Salmonella-typhimurium*-induced colitis [65]. In addition, the abundance of the probiotics *Ligilactobacillus* and Lactobacillaceae significantly increased after cigarette smoke exposure in our study, which may also be responsible for the decline in *Salmonella* in mice after exposure to cigarette smoke. For example, a study reported that treatment with *Lactobacillus reuteri* R2LC or 4659 reduced inflammation of intestinal mucosa in mice [66]; another study found that the administration of *L. reuteri* F-9-35 produced anti-inflammatory effects in mice with colitis [67]. Of note, our results showed that cigarette smoke exposure resulted in a decrease in the abundance of *Bifidobacterium* and *Faecalibacterium*. Bifidobacteria and *Faecalibacterium* have been reported to have the ability to promote health and have immune regulation functions [50,68,69]. Together, these results indicate that cigarette smoke exposure leads to gut microbial disorder, influencing intestinal inflammation. The liver transcriptome and gut microbiome analysis showed that cigarette smoke exposure led to disorders of both the liver metabolism and gut microbiota. In this study, the mechanism of cigarette smoke exposure on health damage in mice was further analyzed from the perspective of the interaction between the gut microbiota and liver gene expression. A significant correlation between intestinal bacterial abundance and liver gene expression after cigarette smoke exposure was detected. For example, the abundance of the harmful bacterium *Salmonella* was negatively correlated with the PPAR-upregulated genes *Acsl3* and *Me1* and positively correlated with the PPAR-downregulated genes *Angptl4*, *Cyp4a12a*, *Plin4*, and *Plin5*. The abundance of the beneficial bacterium *Ligilactobacillus* showed opposite trends of association with these genes. PPARs are abundant in the liver and adipose tissue, participate in a variety of physiological processes, and play an important role in energy homeostasis, lipid metabolism, inflammation, and immune regulation [70,71,72]. These results show that the PPAR signaling pathway could also be regulated by gut microbiota. A similar result was also obtained in a previous study, which demonstrated that the administration of probiotic *Lactobacillus plantarum* activated peroxisome PPARα [73]. Together, these results show that the changes in the gut microbiota of mice treated with cigarette smoke were closely related to liver gene expressions and functions.

## 4. Materials and Methods

### 4.1. Mice and Treatment

It was reported that male animals were more sensitive than females to the discriminative stimulus effects of nicotine [74]. In addition, the number of women using tobacco worldwide declined to 244 million in 2018, while the number of men smoking increased to 1.093 billion in 2018 (82% of the world’s current 1.337 billion tobacco users), according to the WHO global report on trends in prevalence of tobacco use 2000–2025 (third edition). Therefore, male mice were focused on in this study. Male C57BL/6 mice (6 weeks of age) were purchased from GemPharmatech Co., Ltd., (Nanjing, China; License Number SCXK (SU) 2018-0008) and were housed in a specific pathogen-free (SPF) mouse facility (GemPharmatech Co. Ltd., Nanjing, China) at 23 ± 1 °C, 40–70% humidity, and a 12 h light:12 h dark cycle (lights on at 08:00). Animals were supplied with a normal chow diet and sterile water. Bedding was replaced in all experiments every 7 days. After 2 weeks of acclimatization, the mice were randomly divided into three groups (6 mice per group): (1) the control group with normal air exposure (NC); (2) the group with cigarette smoke exposure (CS); (3) and the group with a combination of cigarette smoke exposure and intragastric administration of nicotine-degrading strain JQ581 (CS-IG).

### 4.2. Bacterial Culture and Preparation

The nicotine-degrading strain *P. putida* JQ581 isolated from sediment from the East China Sea was used in this study [38]. The strain JQ581 was stored in the glycerol tube at −80 °C, plated in LB agar, cultured at 37 °C for 24 h, inoculated in fresh Luria broth (LB) medium, and cultured for another 24 h. The cultures were centrifuged at 4000 rpm/min for 5 min at 20 °C and washed twice with sterile normal saline. The cells were resuspended in sterile normal saline to a final concentration of 5 × 10^9^ mL^−1^.

### 4.3. Cigarette Smoke Exposure and Intragastric Administration Treatment

For treatment with cigarette smoke exposure, the mice in the CS and CS-IG groups were placed in a smoke exposure chamber (54 cm long, 33 cm wide, and 17 cm high; Figure S1), exposed to 2 cigarettes (each cigarette contains 1.0 mg of nicotine) at an air-flow rate of 3 L/min for 20–30 min at a time, and exposed three times a day. The flow rate and treatment time were determined based on our pre-experiments, which showed that the mice could keep breathing well at the flow rate of 3 L/min. Then, we explored the time from lighting cigarettes to complete extinction and smoke dissipation. We found that the cigarettes were burnt out in 5–6 min at the flow rate of 3 L/min, and then the smoke in the chamber dissipated in 10 min. After that, the chamber still had a strong smoke smell, so we kept the mice in the chamber for 10–15 min before taking them out. The cigarettes were purchased from Hongta Tobacco Co., Ltd. (Yuxi, China). The mice in the NC group were also placed in the same chamber without cigarette smoke exposure. The treatment lasted for 12 weeks, as significant changes in physiological indexes were detected between the treated and control mice at 12 weeks. Mice were intragastrically administered normal saline without bacteria (NC and CS group, *n* = 6) or with strain JQ581 (1 × 10^9^ cfu; CS-IG group, *n* = 6). This procedure was performed daily for a period of 12 continuous weeks. During the experiments, body weight and food intake were measured every two weeks in the morning. In addition, blood glucose was measured every four weeks by tail vein blood collection after the mice were fasted for 6 h. Fecal samples were collected at the end of the treatment and stored at −80 °C for further analysis. After the mice were anesthetized with ketamine, blood samples were collected from the heart and centrifuged for 10 min at 5000 rpm and 4 °C to obtain serum, which was then stored at −80 °C for further analysis. Then, the mice were euthanized by cervical dislocation, and the colon contents, cecal contents, and liver tissue were collected, immediately frozen in liquid nitrogen, and stored at −80 °C until further analysis.

### 4.4. Physiological and Biochemical Indicators and Histopathology Analysis

The fasting blood glucose of mice was detected with a Roche Accu-Chek Active Glucose Meter. The contents of TC, TG, LDL-C, and HDL-C in serum samples and the activities of serum AST, ALT, and TBIL were determined using a Hitachi 7020 automatic clinical chemistry analyzer (Hitachi, Tokyo, Japan). The colon, cecum, lung, and liver samples of four mice randomly selected from each group were used for histological analysis. Samples were fixed in 4% paraformaldehyde (PFA) overnight, embedded in paraffin, cut into 3–5 μm thick sections, and then stained with H&E.

### 4.5. 16S rRNA Sequencing and Bioinformatics Analyses

The bacterial DNA was extracted from samples of feces, colon, and cecal contents with an E.Z.N.A.^®^ Stool DNA Kit (Omega Bio-tek, Norcross, GA, USA) according to the manufacturer’s protocol. The V3-V4 region of 16S rRNA (forward primer 314F 5′- CCTAYGGGRBGCASCAG-3′ and reverse primer 806R 5′- GGACTACNNGGGTATCTAAT-3′) was amplified through PCR. The PCR-amplified products were detected and purified by 2% agarose gel electrophoresis, and fluorescence quantification was performed with a QuantiFluor™-ST blue fluorescence quantitative system (Promega). PE250 libraries were then generated on the Illumina PE250 platform for sequencing (Shanghai Biozeron Biotechnology Co. Ltd., Shanghai, China). The reads were denoised using DADA2 [75] and clustered into amplicon sequence variants (ASVs) using QIIME2 [76]. A rarefaction analysis based on Mothur v.1.21.1 [77] was conducted to reveal the diversity indices, including the Chao1, ACE, and Shannon diversity indices. A PCoA was performed using the ‘vegan’ package of R v.4.0.2 based on unweighted UniFrac distances [78]. For the identification of biomarkers, a LEfSe (linear discriminant analysis effect size) analysis was performed, and the threshold for the logarithmic linear discriminant analysis (LDA) score for discriminative features was set to 3.0 [79].

### 4.6. Liver Transcriptome Analysis

Total RNA was extracted from the liver tissue using TRIzol^®^ Reagent according to the manufacturer’s instructions (Invitrogen, Carlsbad, CA, USA). The quality of the RNA was determined using a 2100 Bioanalyzer (Agilent, Santa Clara, CA, USA) and then checked via RNase free agarose gel electrophoresis. Transcriptome libraries of the RNA samples were constructed according to the manufacturer’s instructions. Finally, RNA sequencing was performed with an Illumina NovaSeq 6000 (Shanghai Biozeron Biotechnology Co. Ltd., Shanghai, China). A PCA based on gene expression was performed using the “prcomp” function in R v.4.0.2. A differential expression analysis was conducted via the R statistical package edgeR [80]. Genes with a false discovery rate (FDR) less than 0.05 and an absolute fold change ≥ 2 were considered DEGs. The GO enrichment analysis and KEGG pathway enrichment analysis were performed based on DEGs with *p* < 0.05.

### 4.7. Effects of Nicotine on the Growth of Strain LM1

The feces and contents of the colons and cecums of mice were suspended in phosphate-buffered saline (PBS) buffer with 0.1% cysteine, and the large, insoluble particles in the suspension were removed using a cell strainer. The suspension was further gradiently diluted to different concentrations, and 100 μL of each dilution was plated onto Wilkins–Chalgren anaerobe medium or Modified Gifu Anaerobe Medium (MGAM). The plates were incubated at 37 °C under aerobic or anaerobic conditions. Single colonies were streaked at least three times onto fresh agar plates before transferring into broth media. A pure strain, LM1, was isolated from both fecal and intestinal contents of mice. To classify strain LM1 taxonomically, its 16S rRNA gene sequence was amplified and sequenced. A phylogenetic analysis based on the 16S rRNA gene sequences showed the position of strain LM1 within the genus *Limosilactobacillus* (Figure S7).

The strain LM1 was cultured with de Man, Rogosa, and Sharpe (MRS) broth at 37 °C under anaerobic conditions. The cell cultures (OD_600_ = 1.0) of LM1 were transferred to fresh MRS broth with different concentrations of nicotine (0, 0.1, 0.5, 0.8, and 1.0 mg/L) with inoculation proportions of 0.5%. The growth of the strain was monitored by measuring the optical density at 600 nm (OD_600_).

### 4.8. Statistical Analysis

All values are expressed as means ± SEM. Statistical analyses were performed with SPSS (Version 26.0, IBM, Armonk, NY, USA) using one-way ANOVA and Duncan’s test, and differences were considered statistically significant at *p* < 0.05. GraphPad Prism v.8.0 and R v.4.0.2 were applied to visualize the data.

## Data Availability

Raw reads for 16S rRNA gene sequencing and transcriptome were deposited into the NCBI Sequence Read Archive (SRA: PRJNA851097 for 16S rRNA gene sequencing; SRA: PRJNA851478 for transcriptome sequencing) database.

## Supplementary Materials

The following supporting information can be downloaded at: https://www.mdpi.com/article/10.3390/ijms231911008/s1.
