# Supplementary material for: Effects of Cigarette Smoke Exposure on the Gut Microbiota and Liver Transcriptome in Mice Reveal Gut–Liver Interactions
Source: Int J Mol Sci. 2022 Sep 20;23(19):11008. doi: 10.3390/ijms231911008 (PMC9569613; doi:10.3390/ijms231911008)
Supplement: Supplementary file 1 [file ijms-23-11008-s001.zip › ijms-1916009-supplementary.pdf]

Supplementary Figures

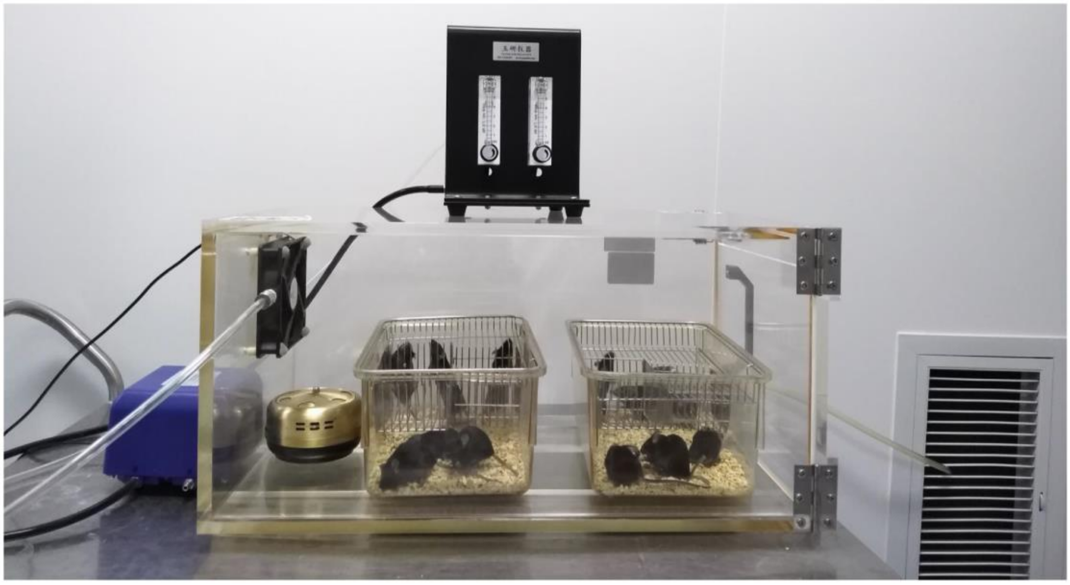

Figure S1. Cigarette smoke exposure devices. The transparent box is a smoking exposure chamber, and the blue device is an air pump to feed fresh air into the chamber. On the chamber is a flowmeter to control the flow rate. The moxibustion box inside the smoking exposure chamber is used to light cigarettes.

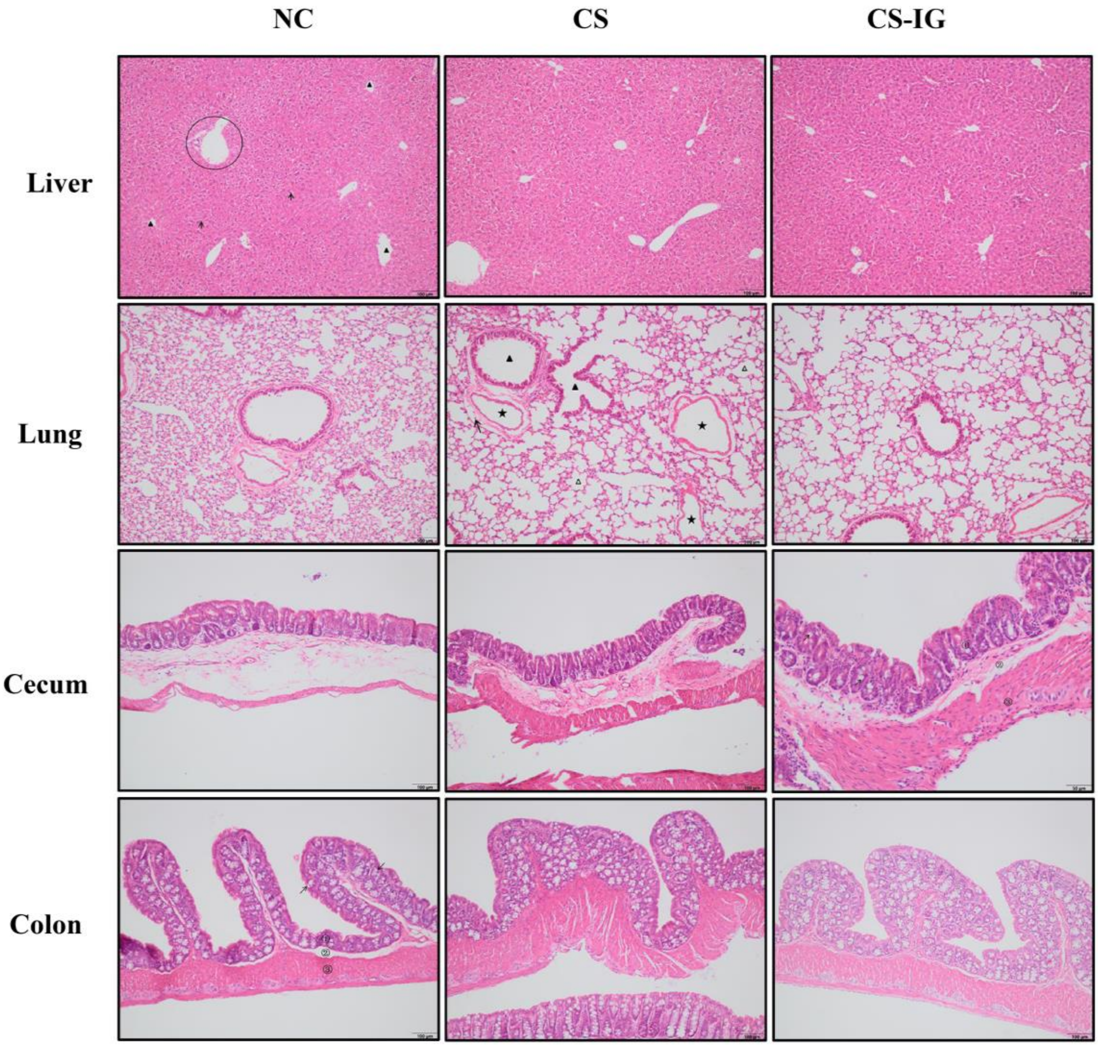

Figure S2. Effects of cigarette smoke exposure and intragastric administration bacteria treatment on the histological and morphological structures of the liver, lung, cecum and colon of mice: representative images of H&E staining of liver, lung, cecum, colon tissues from different groups. Lung: bronchioles (▲); Blood vessel (★); Alveoli (△); Pulmonary vascular space increased (↑); Liver: liver cell (↑); Central vein (▲); Gate area (○); Intestine: Mucosa (①); Submucosa (②); Muscular layer (③); Goblet cell (↑). NC, the mice treated with normal air exposure; CS, the mice treated with cigarette smoke exposure; CS-IG group, the mice treated with combination of cigarette smoke exposure and intragastric administration of nicotine-degrading strain JQ581.

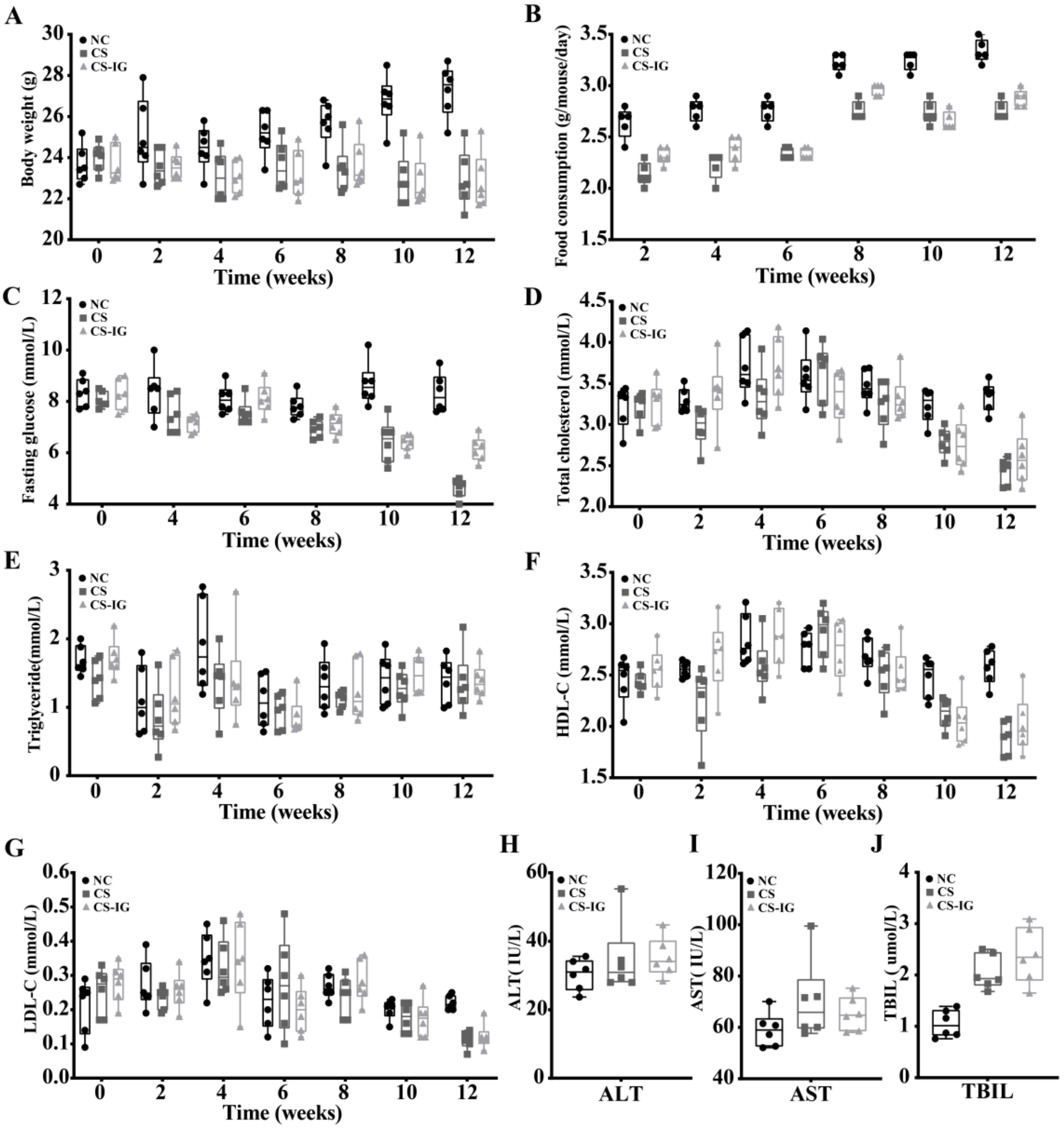

Figure S3. Effects of cigarette smoke exposure on the physiological and biochemical indicators of mice. (A) Body weight. (B) Food consumption. (C) Fasting glucose. (D) Serum total cholesterol. (E) Serum triglyceride. (F) Serum HDL-C. (G) Serum LDL-C. (H-J) AST (H) and ALT (I) activities and TBIL (J) contents in blood serum. LDL-C, low-density lipoprotein cholesterol; HDL-C, high-density lipo-protein cholesterol; AST, aspartate aminotransferase; ALT, alanine aminotransferase; TBIL, total bilirubin. NC, the mice treated with normal air exposure; CS, the mice treated with cigarette smoke exposure, CS-IG, the mice treated with combination of cigarette smoke exposure and intragastric administration of nicotine-degrading strain JQ581. Different letters indicate significant differences (Duncan's test, n=6, $P < 0.05$).

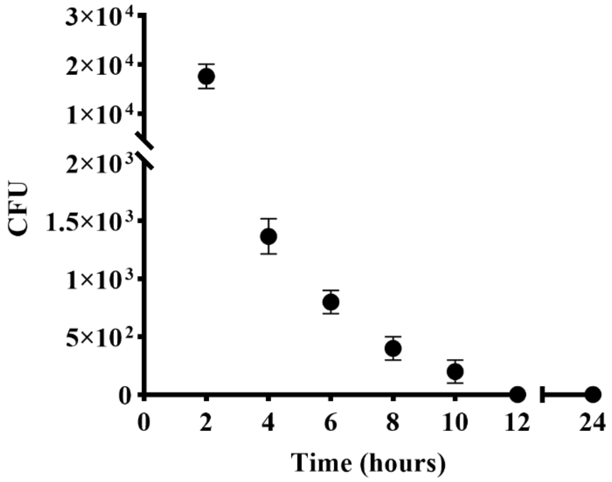

Figure S4. Isolation of *Pseudomonas putida* JQ581 from the fecal samples of mice from CS-IG group at different times after treatment of intragastric administration. Data were expressed as means ± SEM (n=6).

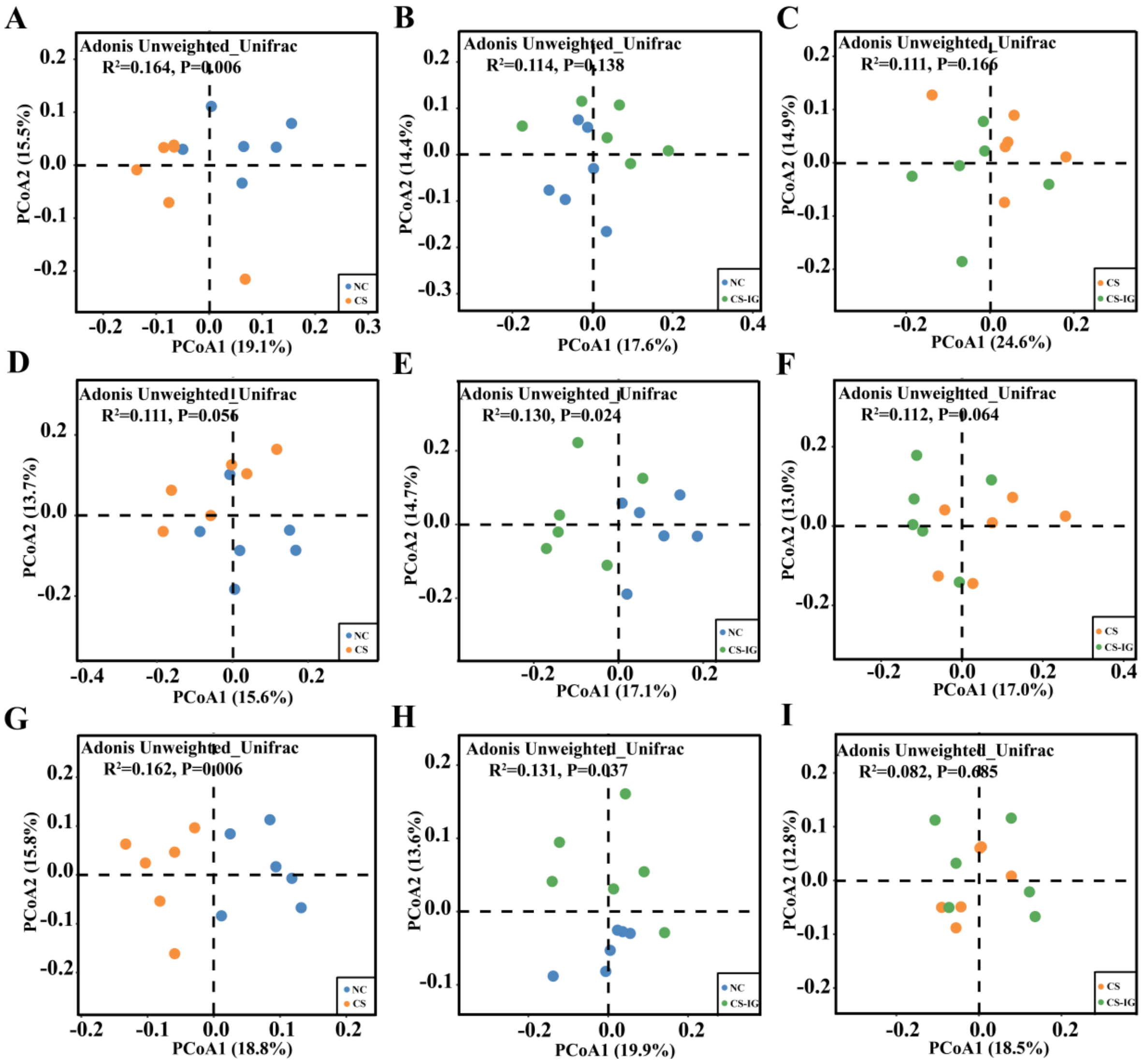

Figure S5. The effects of cigarette smoke exposure and intragastric administration of JQ581 on the structure of gut microbiota at 12 weeks after treatments. Principal coordinate analysis (PCoA) based on ASVs of feces (A-C), colon (D-F) and cecal contents (G-I) in mice are showed. NC, the mice treated with normal air exposure; CS, the mice treated with cigarette smoke exposure; CS-IG, the mice treated with combination of cigarette smoke exposure and intragastric administration of nicotine-degrading strain JQ581.

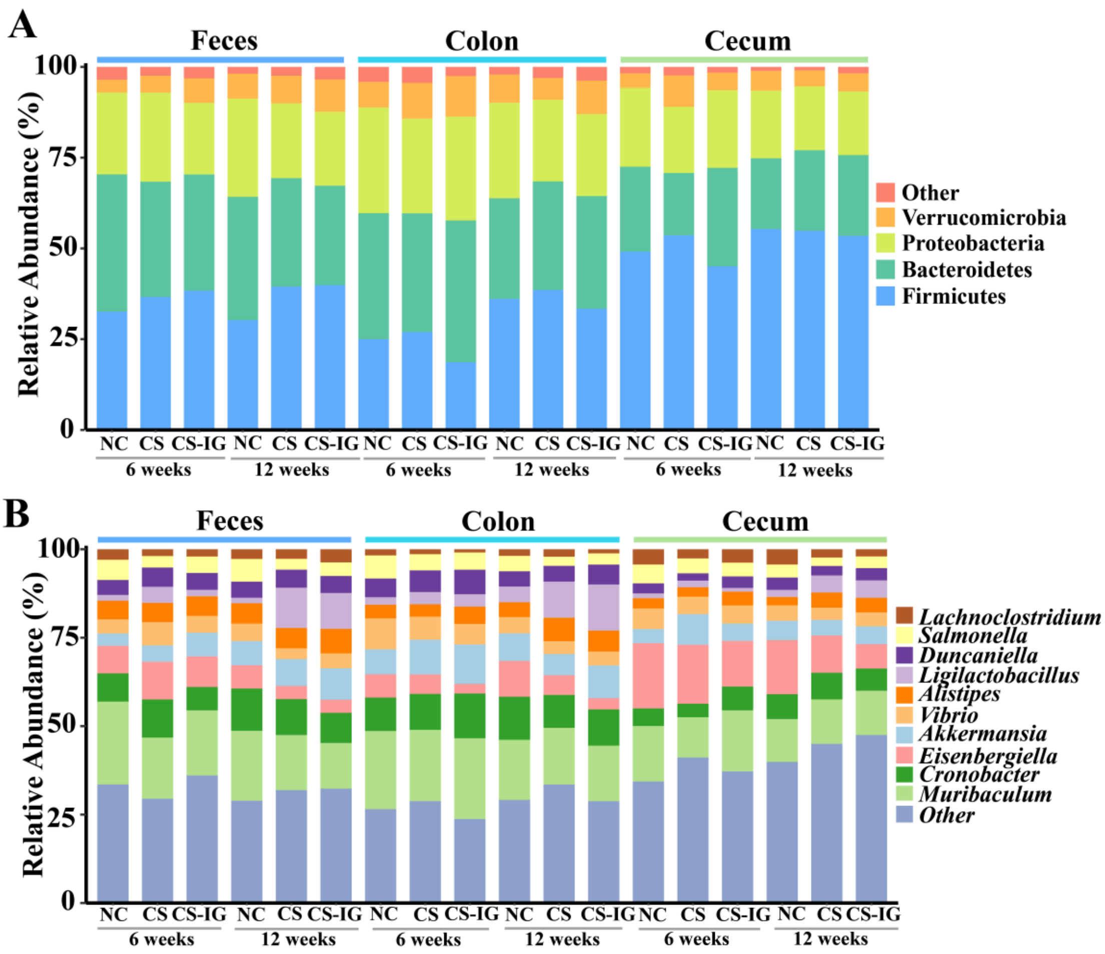

Figure S6. The predominant taxa in gut microbiota. (A-B) Average relative abundances of gut microbiota constituents at the phylum (A) and genus (B) level in mice. NC, the mice treated with normal air exposure; CS, the mice treated with cigarette smoke exposure; CS-IG, the mice treated with combination of cigarette smoke exposure and intragastric administration of nicotine-degrading strain JQ581.

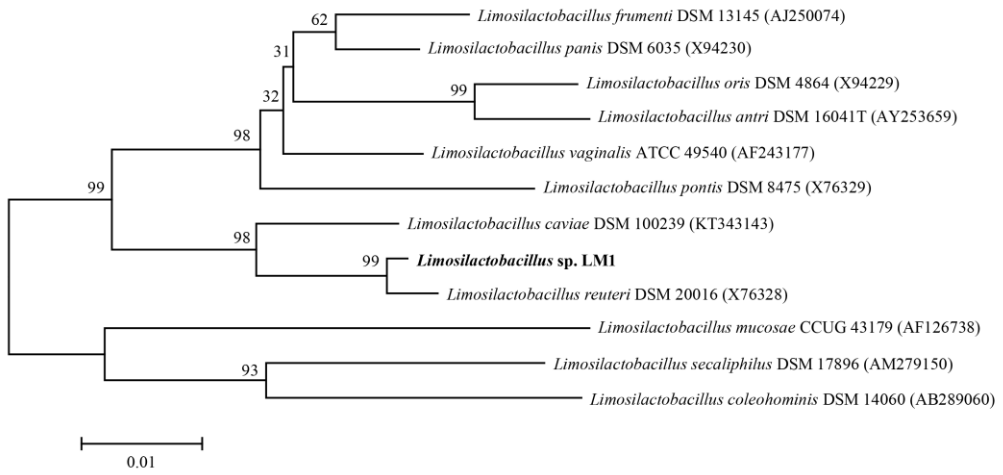

Figure S7. Phylogenetic analysis of strain LM1 and other representatives of the genus *Limosilactobacillus* based on 16S rRNA gene sequences. The 16S rRNA gene sequences of strain LM1 and other related strains were aligned and manually adjusted using CLUSTALW. Phylogenetic trees were constructed based on neighbor-joining method with 1,000 replications using MEGA v7.0. The bootstrap values based on 1,000 replications are indicated above the branches. Bar, 0.01 substitutions per nucleotide position.

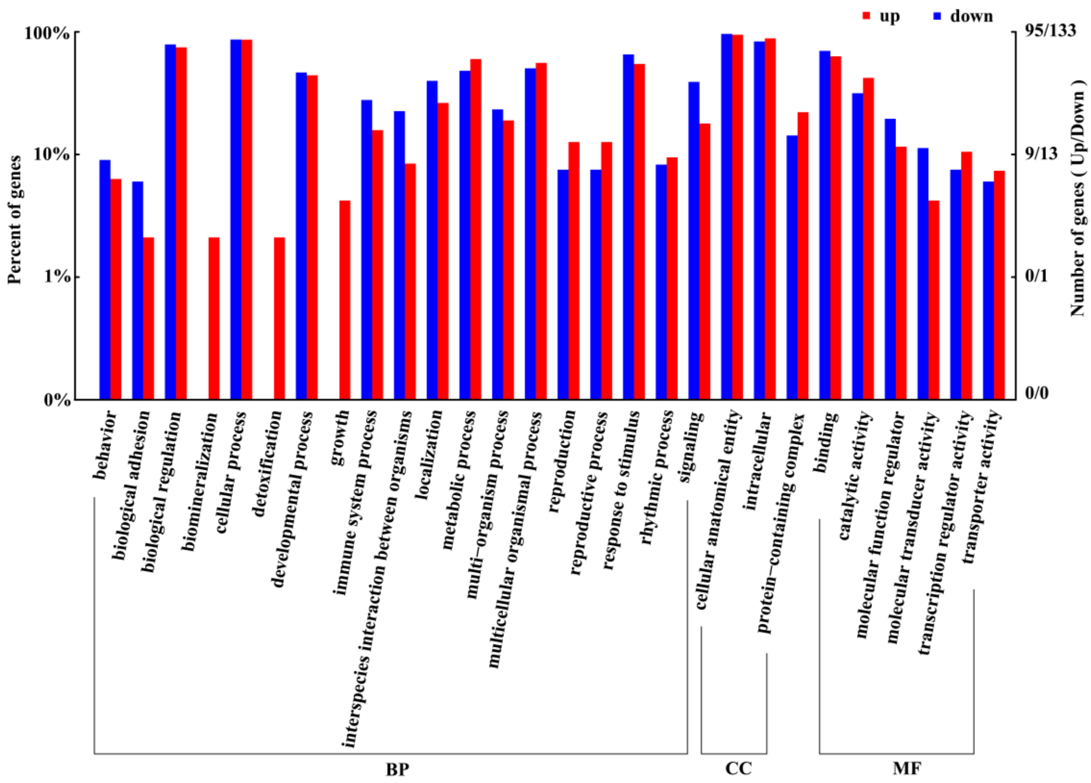

Figure S8. Histogram of GO annotations of the DEGs between the NC and CS groups. BP, biological processes; CC, cellular components, MF: molecular functions. NC, the mice treated with normal air exposure; CS, the mice treated with cigarette smoke exposure.

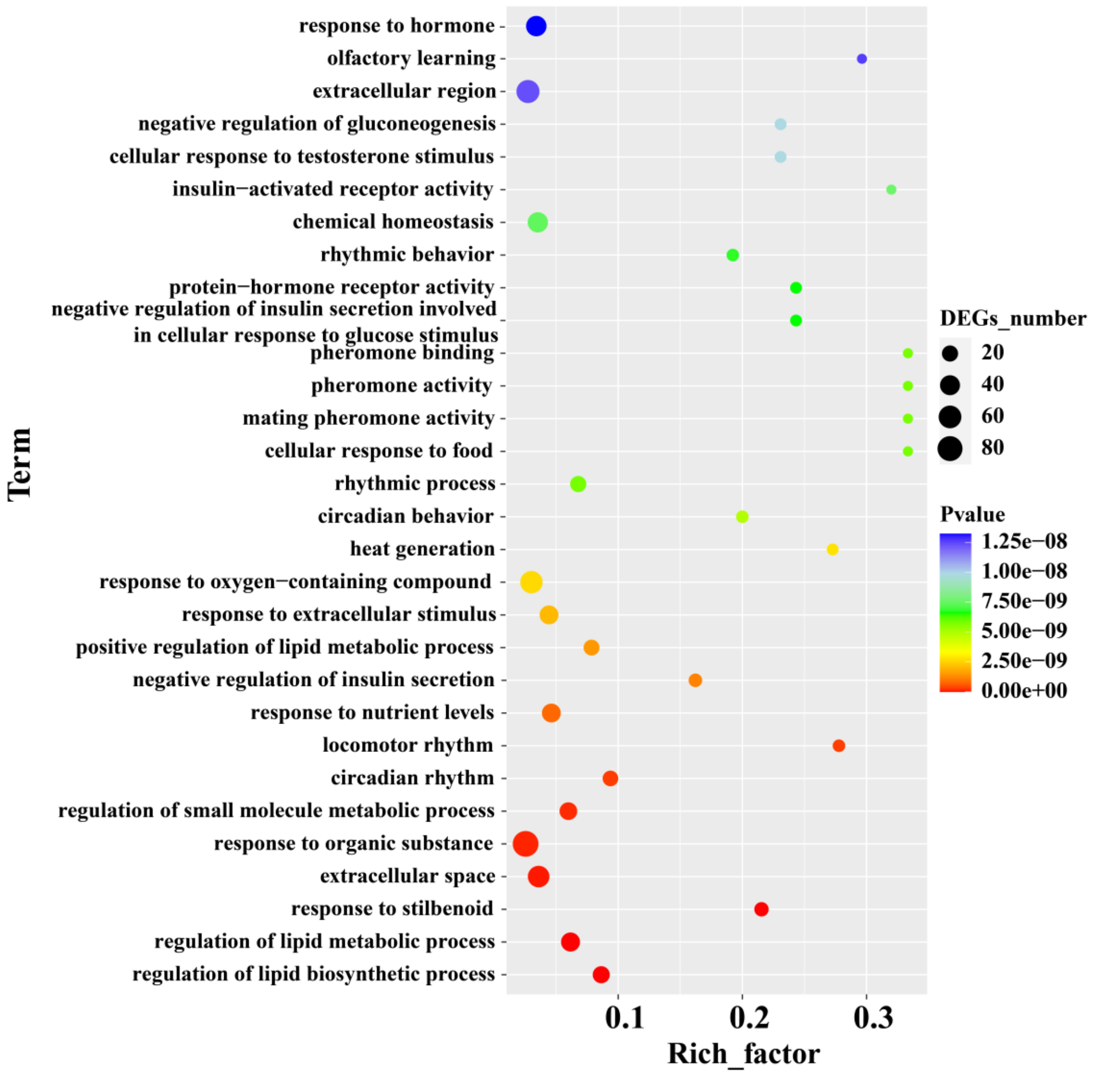

Figure S9. GO enrichment map of DEGs of the top 30.

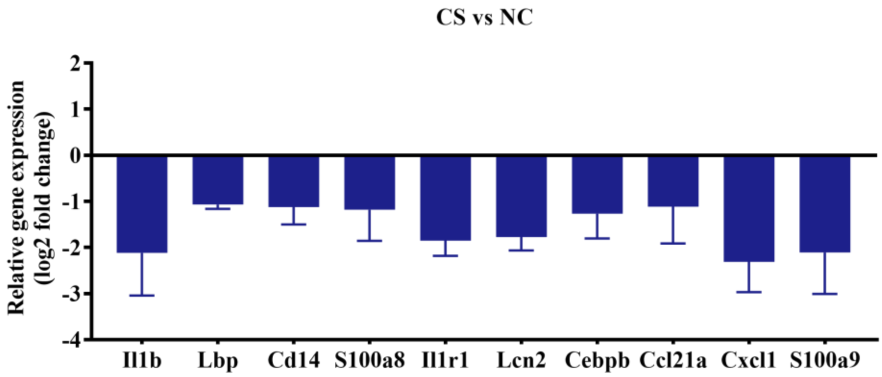

Figure S10. Down-regulation of gene expressions involved in immune response, signal transduction, and inflammation pathway by cigarette smoke exposure.

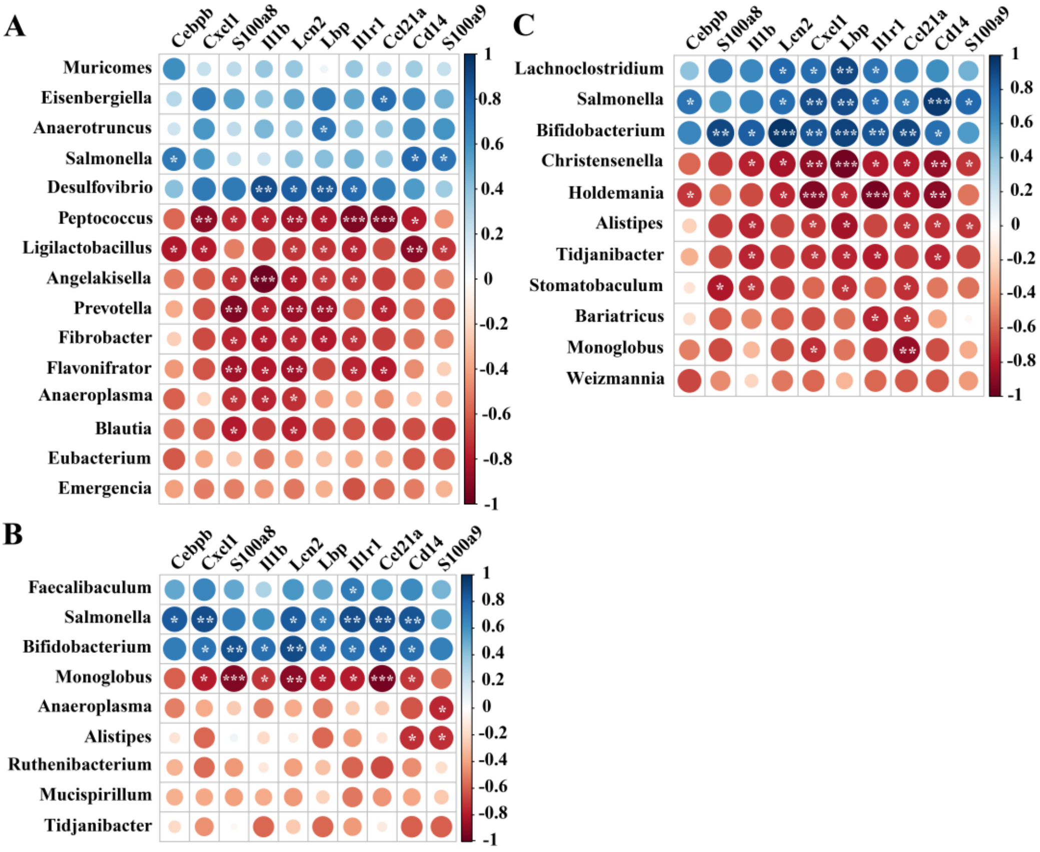

Figure S11. Interactions between gut microbiota and expressions of genes related to immune response, signal transduction, and inflammation in liver. (A-C) Spearman correlations of gut microbiota from feces (A), colon (B), and cecal contents (C) with these genes, respectively. *p<0.05, **p<0.01, ***p<0.001, n=4.

Supplementary Tables

Table S1. α-diversity index

Sample	Time (weeks)	Group	ACE	Chao1	Shannon	Coverage
Feces	0	NC	346.83±46.78 (abc)	346.83±46.78 (abc)	6.42±0.42 (abc)	1
		CS	346.83±63 (abcd)	334.83±50.69 (abcd)	6.39±0.39 (abc)	1
		CS-IG	317.33±61.29 (abcde)	317.33±48.59 (abcde)	6.35±0.23 (abc)	1
	6	NC	370±44.67 (a)	370±44.67 (a)	6.64±0.17 (a)	1
		CS	370±37.28 (ab)	362.33±43.8 (ab)	6.33±0.29 (abc)	1
		CS-IG	355.33±31.43 (ab)	355.33±36.81 (ab)	6.66±0.34 (a)	1
	12	NC	283.33±39.19 (cde)	283.33±39.19 (cde)	6.37±0.3 (abc)	1
		CS	283.33±40.04 (abcd)	322.83±67.97 (abcd)	6.48±0.33 (abc)	1
		CS-IG	295.5±43.04 (bcde)	295.5±67.34 (de)	6.33±0.53 (abc)	1
Colon	6	NC	283.83±27.91 (cde)	283.83±27.91 (cde)	6.34±0.25 (abc)	1
		CS	283.83±40.24 (de)	276.5±65.57 (de)	6.17±0.3 (abc)	1
		CS-IG	254.67±38.17 (e)	254.67±46.36 (e)	6.03±0.36 (c)	1
	12	NC	346.33±38.82 (abc)	346.33±38.82 (abc)	6.48±0.31 (abc)	1
		CS	346.33±37.01 (ab)	362.33±18.64 (ab)	6.6±0.13 (ab)	1
		CS-IG	360.17±38.01 (ab)	360.17±30.83 (ab)	6.54±0.33 (abc)	1
Cecum	6	NC	306.33±44.38 (abcde)	306.33±44.38 (abcde)	6.18±0.73 (abc)	1
		CS	306.33±29.15 (cde)	280.67±42.37 (cde)	6.06±0.48 (bc)	1
		CS-IG	308.17±28.63 (abcde）	308.17±31.32 (abcde）	6.29±0.3 (abc)	1
	12	NC	285±34.75 (cde)	285±34.75 (cde)	6.07±0.39 (bc)	1
		CS	285±38.44 (abcde)	304±28.9 (abcde)	6.41±0.26 (abc)	1
		CS-IG	316.83±34.38 (abcde)	316.83±45.99 (abcde)	6.24±0.35 (abc)	1

Value are means followed by SEM. Different letters indicated statistically significant differences (P < 0.05) according to the Duncan's test.

Table S2. Comparisons of liver transcriptome sequencing data among NC, CS, and CS-IG groups.

Sample	Raw reads	Clean reads	Mapped reads	Mapped rate(%)	Q30	GC(%)
NC-1	88620934	76376340	69899626	91.52	93.17	49.00
NC-2	67754548	61103124	54864495	89.79	92.21	48.84
NC-3	72251746	64299676	58763473	91.39	93.1	48.73
NC-4	79404036	70361748	64697627	91.95	92.68	49.10
CS-1	64935566	58975986	54653046	92.67	92.61	48.19
CS-2	72395904	63856684	58799234	92.08	92.96	49.49
CS-3	38216240	32492142	29519111	90.85	92.45	48.46
CS-4	48005860	42698812	38458819	90.07	92.72	48.10
CS-IG-1	56254210	50698286	46581585	91.88	91.99	48.98
CS-IG-2	34749574	30703222	27715798	90.27	92.45	48.54
CS-IG-3	62252350	54947470	48837311	88.88	92.97	47.62
CS-IG-4	57104656	49739712	43606805	87.67	93.08	48.62
